# A Retrospective Single-Center Comparative Study Between Robot-Assisted and Laparoscopic Radical Nephroureterectomy for Upper-Tract Urothelial Carcinoma on Perioperative Results, Overall Survival, and Recurrence Rate

**DOI:** 10.7759/cureus.66623

**Published:** 2024-08-11

**Authors:** Saleh Al-Gburi, Omer Abdalla

**Affiliations:** 1 Urology, Wirral University Teaching Hospitals, Wirral, GBR; 2 Urology, Mosul Medical College, University of Mosul, Mosul, IRQ

**Keywords:** nephroureterectomy, lap surgery, robotic surgical procedures, transitional cell carinoma, upper urinary tract

## Abstract

Introduction

Patients with localized high-risk urothelial carcinoma of the upper urinary tract are advised radical nephroureterectomy, the surgical removal of the kidney and ureter, utilizing robot-assisted versus laparoscopic methods. This study aims to compare the surgical and oncological results of robot-assisted and laparoscopic radical nephroureterectomy for upper-tract urothelial carcinoma.

Methods

An observational retrospective cohort study compared 14 patients who had robotic-assisted nephroureterctomy (RAN) to 16 patients who had laparoscopic assisted nephroureterctomy (LAN).

Results

There was no significant difference in age, sex, glomerular filtration rate (GFR), creatinine, Charlson comorbidity score, length of hospital stays, or the need to convert to an open approach. However, there was a statistical difference between the two procedures in terms of lymph dissection (p-value of 0.037) and the length of the procedure (p-value of 0.09).

Conclusions

The robotic approach has significantly higher use for lymph node dissection, while laparoscopic radical nephroureterectomy has a shorter operation time.

## Introduction

Urothelial carcinoma of the upper tract constitutes a mere 5% of the total urothelial tumors [1]. Approximately 60% of these tumors are located in the pelvicalyceal system, whereas the remaining tumors are situated along the ureter [2].

Over time, the evaluation and care of upper urinary tract urothelial cancer patients have advanced, and a multi-disciplinary approach is necessary to maximize the cancer-related results for a condition that nevertheless poses a significant risk of mortality [3]. One may consider using a robot-assisted laparoscopic method, which offers similar perioperative advantages as normal laparoscopic surgery [4].

Surgical removal of the kidney and ureter, known as radical nephroureterectomy, is recommended for individuals with localized high-risk disease. Laparoscopic radical nephroureterectomy (LAN) is considered safe when performed by qualified surgeons who strictly follow oncological standards. The popularity of the robotic-assisted method has increased recently due to the convenience of removing the bladder cuff and performing lymphadenectomy [5].

Our study aimed to assess and compare the perioperative results of laparoscopic and robotic surgeries in patients with localized, non-metastatic upper urinary tract cancer. Additionally, we aimed to investigate the relationship between each surgical strategy, the rate of recurrence, metastasis-free survival, and overall survival.

## Materials and methods

Study design and patient selection

In an observational retrospective cohort study, we evaluated data from 14 patients who had robotic-assisted nephroureterctomy (RAN) and compared it to 16 patients who had laproscopic-assisted nephroureterctomy (LAN). We excluded every patient who had a concurrent or previous diagnosis of bladder cancer; some patients had their follow-up done in other hospitals, therefore excluded from the study. Patients who had metastatic disease or incomplete data were also excluded from the study.

General demographic, clinical, pathological, perioperative, and oncology details were collected retrospectively. A comparative analysis was conducted between the robotic and laparoscopic techniques of nephroureterectomy.

Statistical analysis

Categorical data is presented in the form of frequencies and percentages, whereas continuous variables are presented using means and standard deviations, or medians.

Univariate analysis was done as the following continuous variables were compared using either a t-test or a Mann-Whitney test, depending on whether the variables had a normal or skewed distribution, respectively. The comparison of categorical variables was conducted using either Fisher's exact test or the chi-square test, depending on the specific conditions. The Shapiro-Wilk test was employed to assess the normality of the data.

Survival curves using the Kaplan-Meier method were computed for each surgical strategy. The log-rank (Mantel-Cox) test was used to determine the difference or equivalence between treatment groups. Patients who did not experience the event were censored at their last follow-up date. The significance level was established at a threshold of p < 0.05.

IBM Corp. Released 2017. IBM SPSS Statistics for Windows, Version 25.0. Armonk, NY: IBM Corp. was used to conduct statistical analyses.

Robotic nephroureterectomy was done using the Da Vinci Xi robotic system. Bladder cuff excision was done either endoscopically or open procedure.

The follow-up evaluation consisted of a comprehensive assessment that involved obtaining the patient's medical history, physical examination, and laboratory tests, including a complete blood count, kidney and liver function tests, urine routine, and urine cytology. In addition, diagnostic procedures such as cystoscopy, ultrasound, or fluorodeoxyglucose positron emission tomography (FDG PET) scan were also conducted. The follow-up regimen consisted of appointments every three months during the initial two-year period, every six months for the subsequent three years, and annually thereafter.

## Results

A total of 30 patients were included in this study; all patients were operated on by experienced surgeons; 16 patients were operated on laparoscopically, and 14 patients were operated on robotically. There was no statistical significance in age, sex, GFR, creatinine, or Charlson comorbidity score (Table 1). 

**Table 1:** General clinical features N: Number, LAN: Laparoscopic nephrouretectomy, RAN: Robotic nephrouretectomy

Table *1*	LAN the number (N) = 16	RAN the number (N) = 14	p-value
Age (mean +/- SD)	71 +/- 9	70 +/- 6	0.886
Sex			0.220
Male N (%)	14 (87.5%)	10 (71%)	
Female N (%)	2 (12.5%)	4 (28.5%)	
GFR (mean +/- SD)	63 +/-16	53 +/-12.5	0.053
Pre op creatinine (mean +/- SD)	104 +/- 25	116 +/- 28	0.255
Charlson comorbidity score (mean +/- SD)	5 +/- 1.4	4.79 +/- 0.802	0.45

Regarding pathological characteristics, there was no statistically significant difference regarding the side, location, or use of adjuvant chemotherapy (Table 2).

**Table 2:** Pathological characteristics N: Number, LAN: Laparoscopic nephrouretectomy, RAN: Robotic nephrouretectomy

Table *2*	LAN N = 16	RAN N = 14	p-value
Grade			
1	1 (6.2%)	2 (14.2%)	
2	6 (37.5%)	5 (35.7%)	
3	9 (56.2.%)	7 (50 %)	
T stage			
Ta	5 (31%)	7 (50%)	
T1	2 (12%)	1 (7%)	
T2	4 (25%)	3 (21%)	
T3	5 (31%)	3 (21%)	
Laterality			0.99
Right N (%)	6 (38%)	5 (36%)	
Left N (%)	10 (62%)	9 (64%)	
Location			0.704
Renal pelvis N (%)	6 (38%)	4 (28%)	
Ureter N (%)	10 (62%)	10 (72%)	
Adjuvant chemotherapy N (%)	1 (5%)	4 (28%)	0.394

The laparoscopic group had a positive surgical margin in two patients (12.5%), and the robotic group had a positive surgical margin in two patients (14%) as well. The difference between the two groups was not statistically significant (p = 0.57). There was no statistically significant difference between the two procedures in terms of length of hospital stay or the need to convert to an open approach. 

In the laparoscopic group, lymph node resection was performed in one patient (6%), while in the robotic group, lymph node resection was performed in five patients (35%). The distinction between the two groups was statistically significant, with a p-value of 0.037. The mean operation time in the laparoscopy approach was 185 minutes, while it was 237 minutes in the robotic approach, and it was statistically significant with a p-value of 0.09 (Table 3).

**Table 3:** Surgical characteristics of the procedures N: Number, LAN: laparoscopic nephrouretectomy, RAN: robotic nephrouretectomy

Table *3*	LAN N = 16	RAN N = 14	p-value
Lymph node resection N (%)	1 (6%)	5 (35%)	0.037
Converted to open	2 (12.5%)	2 (14%)	0.57
Operation time (mean +/- SD) (minutes)	185 (37)	237 (72)	0.09
Hospital admission median (days)	5.5	4	0.313
Positive Resection margin	2 (12.5%)	2 (14%)	0.57
Post op complications	None	None	
Need for transfusion	None	None	

Kaplan-Meier survival analysis was conducted to compare the two operations for their effect on the treatment of upper urinary tract urothelial cancer. A log-rank test (Mantel-cox) was conducted to determine if there were differences in survival distributions for the operations, which was 0.269.

**Figure 1:**
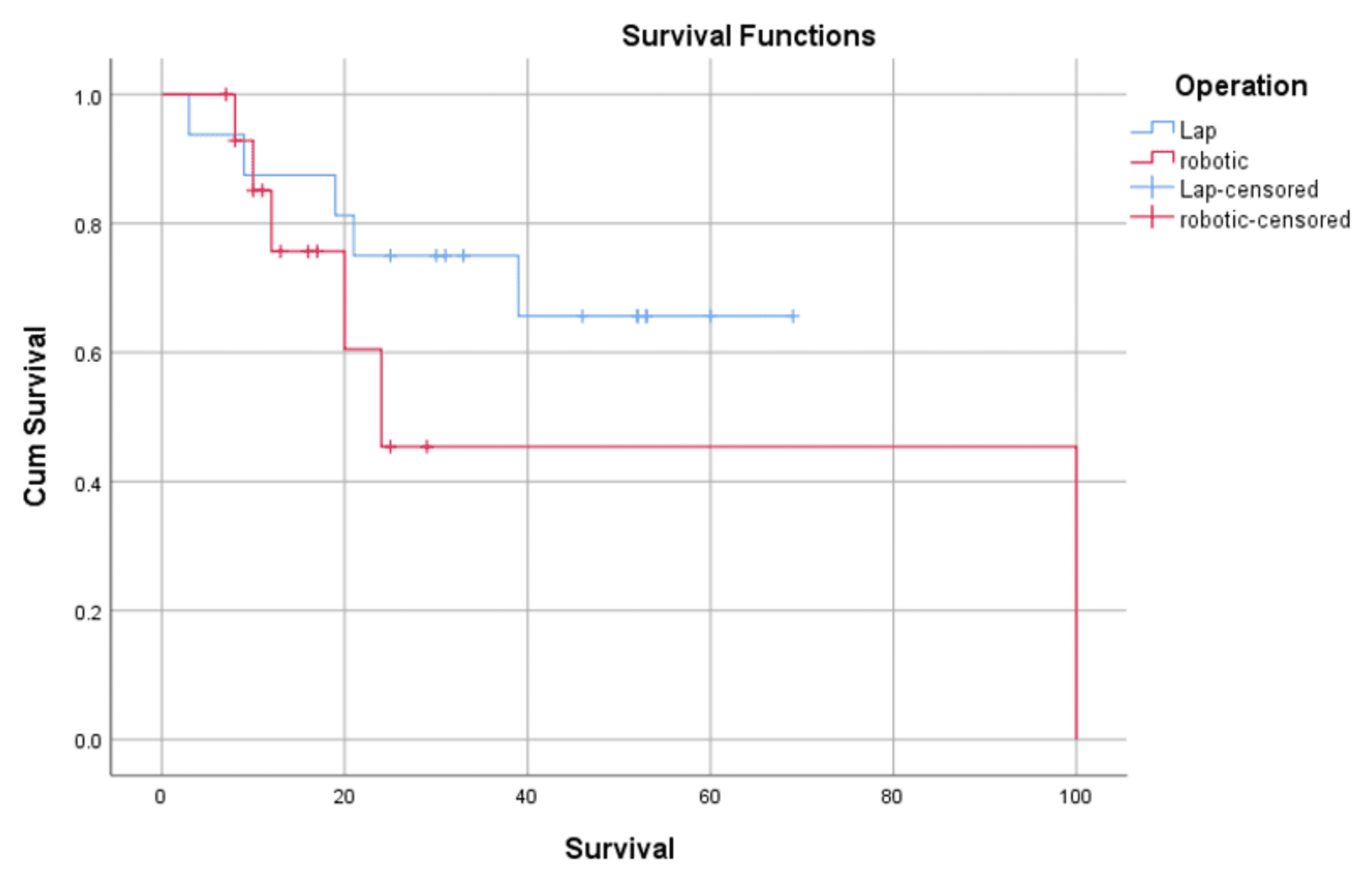
Kaplan-Meier estimates of metastasis-free survival

There was no significant change regarding survival between the two operations (Figure 1).

Overall recurrences were observed in 10 patients (62% of total patients) in the laparoscopic group compared to eight patients (57% of total patients). The median duration for overall recurrence was seven months in both groups (Table 4).

**Table 4:** Overall recurrence and mortality of the procedures N: Number, LAN: Laparoscopic nephrouretectomy, RAN: Robotic nephrouretectomy

Table *4*	LAN N = 16	RAN N = 14	p-value
Median follow up in months (range)	36 (3-69)	18 (7-100)	0.006
Overall recurrence	10 (62%)	8 (57%)	0.074
Median time in recurrence (range)	7 (1-19)	7 (1-92)	0.833
Overall mortality	5 (31%)	5 (35%)	

## Discussion

This study examines and contrasts the surgical and oncological results of robotic radical nephroureterectomy with laparoscopic radical nephroureterectomy. The patient populations in both groups were similar in terms of age, kidney function, comorbidities, disease stage, and rates of chemotherapy utilization. The group of robots had elevated rates of lymph node dissection but with longer operation times.

In 2006, Nanigian et al. conducted the first robotic distal ureterectomy, which entailed a laparoscopic nephrectomy and distal excision utilizing the Da Vinci™ system [6]. In 2010, Eandi et al. conducted the first complete RAN. They introduced a lateral rectus port configuration for the first removal of the kidney and ureter, followed by undocking the robot and repositioning the patient for the distal ureterectomy [7].

In 2014, Ambani et al. showed that the perioperative and oncologic outcomes of their initial robotic-assisted nephrouretectomy group were comparable to those for a laparoscopic approach and there were similar complication rates. The duration of the operation and the amount of blood loss were notably higher when employing the robotic-assisted technique [8].

In 2019, Lee et al. conducted a study at a single institution. The study included 422 patients who underwent open, laparoscopic, or robotic procedures for non-metastatic upper urinary tract cancer. The study consisted of 161 patients in the open group, 137 patients in the laparoscopic group, and 124 patients in the robotic group. The findings indicated that RAN and LAN exhibited superior perioperative outcomes in comparison to open surgery. Robotic-assisted surgery demonstrated superior oncologic outcomes in the bivariate analysis, but these benefits were not detected in the multivariate analysis [9].

A literature review by Veccia et al. showed robotic procedures were more commonly conducted in individuals who had tumors located in many areas and had high-grade disease, and the robotic group had the lowest estimated blood loss observed. There was no observed link between the surgical method and the rates of recurrence-free and cancer-specific survival, similar to the findings of our research [10].

Studies have found that laparoscopic RNU has a faster operative duration compared to robotic RNU, probably due to the additional time needed for repositioning and/or redocking of the robot and lymph node dissection, in line with the findings of our research [8,11].

There is a significant amount of evidence indicating that RAN procedures result in a greater number of removed lymph nodes and a higher rate of lymphadenectomy utilization [10,12].

Although the survival benefit of lymphadenectomy in upper urinary tract transitional cell carcinoma is not yet demonstrated, the use of RAN is certain to provide a distinct advantage in terms of accurate staging, improved disease characterization, and improved adjuvant therapy planning [13].

The pattern and risk factors for local recurrence after radical nephroureterectomy were reported by Li et al. [14]. Stonier et al. indicate that the perioperative and oncological performance of these methods is comparable. In addition, there might be a decreased postoperative mortality and total complication rate in the robotic group [15].

In terms of cost, Trudeau et al. reported on comparative costs in their study conducted in the United States. According to their findings, the mean cost was substantially higher in RAN [16].

The present study has significant limitations. First, the series being retrospective introduces a natural bias into the series. In addition, the size of the study is small.

The study's strength also lies in its use of data from a single center. An advantage of having a single center is that all surgeries are conducted by three highly skilled surgeons with a reasonable mean time of follow-up.

## Conclusions

The robotic approach has significantly higher use for lymph node dissection with a longer operation time. The utilization of sophisticated robotic technology enables the treatment of patients with suspected lymph node metastases to be performed more frequently. There was no statistically significant difference in terms of perioperative complications, recurrence rate, or overall survival.

A prospective, randomized trial with sufficient sample size is necessary to establish the position of the robotic approach for upper urinary tract urothelial cancer.

## References

[1] Rouprêt M, Zigeuner R, Palou J (2012). European guidelines for the diagnosis and management of upper urinary tract urothelial cell carcinomas: 2011 update. Actas Urológicas Españolas (English Edition.

[2] Munoz JJ, Ellison IM (2000). Upper tract urothelial neoplasms: incidence and survival during the last 2 decades. Jr Uro.

[3] Kenigsberg AP, Meng X, Ghandour R, Margulis V (2020). Oncologic outcomes of radical nephroureterectomy (RNU). Transl Androl Urol.

[4] Veccia A, Carbonara U, Djaladat H (2022). Robotic vs laparoscopic nephroureterectomy for upper tract urothelial carcinoma: a multicenter propensity-score matched pair “tetrafecta” analysis (ROBUUST collaborative group). J Endourol.

[5] Vasudeo V, Singh A, Khanna A (2023). Surgical and oncological outcomes of robot-assisted versus laparoscopic radical nephroureterectomy for upper-tract urothelial carcinoma: A single-center comparative analysis. Indian J Urol.

[6] Nanigian DK, Smith W, Ellison LM (2006). Robot-assisted laparoscopic nephroureterectomy. J Endourol.

[7] Eandi JA, Nelson RA, Wilson TG, Josephson DY (2010). Oncologic outcomes for complete robot-assisted laparoscopic management of upper-tract transitional cell carcinoma. J Endourol.

[8] Ambani SN, Weizer AZ, Wolf JS Jr, He C, Miller DC, Montgomery JS (2014). Matched comparison of robotic vs laparoscopic nephroureterectomy: an initial experience. Urology.

[9] Lee H, Kim HJ, Lee SE, Hong SK, Byun SS (2019). Comparison of oncological and perioperative outcomes of open, laparoscopic, and robotic nephroureterectomy approaches in patients with non-metastatic upper-tract urothelial carcinoma. PLoS One.

[10] Veccia A, Antonelli A, Francavilla S (2020). Robotic versus other nephroureterectomy techniques: a systematic review and meta-analysis of over 87,000 cases. World J Urol.

[11] Melquist JJ, Redrow G, Delacroix S, Park A, Faria EE, Karam JA, Matin SF (2016). Comparison of single-docking robotic-assisted and traditional laparoscopy for retroperitoneal lymph node dissection during nephroureterectomy with bladder cuff excision for upper-tract urothelial carcinoma. Urology.

[12] Kenigsberg AP, Smith W, Meng X (2021). Robotic nephroureterectomy vs laparoscopic nephroureterectomy: increased utilization, rates of lymphadenectomy, decreased morbidity robotically. J Endourol.

[13] Goltzman ME, Gogoj A, Ristau BT (2020). The role of lymphadenectomy at the time of radical nephroureterectomy for upper tract urothelial carcinoma. Transl Androl Urol.

[14] Li X, Cui M, Gu X (2020). Pattern and risk factors of local recurrence after nephroureterectomy for upper tract urothelial carcinoma. World J Surg Oncol.

[15] Stonier T, Simson N, Lee SM (2017). Laparoscopic vs robotic nephroureterectomy: Is it time to re-establish the standard? Evidence from a systematic review. Arab J Urol.

[16] Vincent T, Giorgio G, Jonas S al. (2014). Robot-assisted versus laparoscopic nephroureterectomy for upper-tract urothelial cancer: A population-based assessment of costs and perioperative outcomes. Cana Uro Assoc Jr.

